# The Emergence of a Psychotic Disorder With a Diagnosis of Breast Cancer Metastasized to the Brain: A Case Report

**DOI:** 10.7759/cureus.49335

**Published:** 2023-11-24

**Authors:** Tuğçe Taşar Yıldırım, Sevler Yıldız

**Affiliations:** 1 Internal Medicine, Fethi Sekin City Hospital, Elazıg, TUR; 2 Psychiatry, Fethi Sekin City Hospital, Elazıg, TUR

**Keywords:** breast oncology, schizophrenia and other psychotic disorders, life-threatening event, metastatic breast cancer, psychotic disorder

## Abstract

It is known that psychotic patients have a reduced ability to evaluate reality and self-care. However, no case has been reported in which a breast lump was misdiagnosed as an insect bite and neglected, and a diagnosis of psychotic disorder led to metastatic breast cancer. A 44-year-old woman diagnosed with invasive breast cancer with brain metastasis became unresponsive with little reaction to verbal communication as a result of successive life-threatening events. After her presentation to the emergency department, she was diagnosed with metastatic breast cancer as a result of detailed examinations. The patient, who did not have any psychiatric illness or alcohol or drug addiction in her medical history, was so insensitive to herself and her environment that she could not notice the large mass in her breast and the bad odors coming from her. According to these findings, the patient was suspected to have a psychotic disorder accompanied by substupor, and olanzapine 2.5 mg/day was administered. If the diagnosis of psychotic disorder is not recognized and treated, the highly visible breast lump may be misperceived and cancer treatment may be delayed, thus the cancer may progress. Early recognition and treatment of mental disorders affect the mortality and morbidity of patients.

## Introduction

Psychotic disorder is a psychiatric disorder in which the person becomes indifferent to the environment and herself [1]. In addition to thought and perception disorders, alienation from the body, inability to recognize bodily changes, and inability to feel pain may be present [2,3]. This is particularly important when you consider that it is associated with reduced quality of life in patients with serious psychiatric disorders [4]. Patients with psychosis are less likely to know or understand their medical problems and are more likely than others to experience serious medical problems due to inadequate medical care [5]. One of the most important diseases affecting morbidity and mortality in these patients is cancer [6,7].

The incidence of breast cancer in patients with psychosis is higher than in the general female population [8]. Here, we describe a patient who, after being diagnosed with advanced breast cancer, was diagnosed with undiagnosed psychosis and experienced a succession of life-threatening events caused by this condition.

## Case presentation

**Figure 1:**
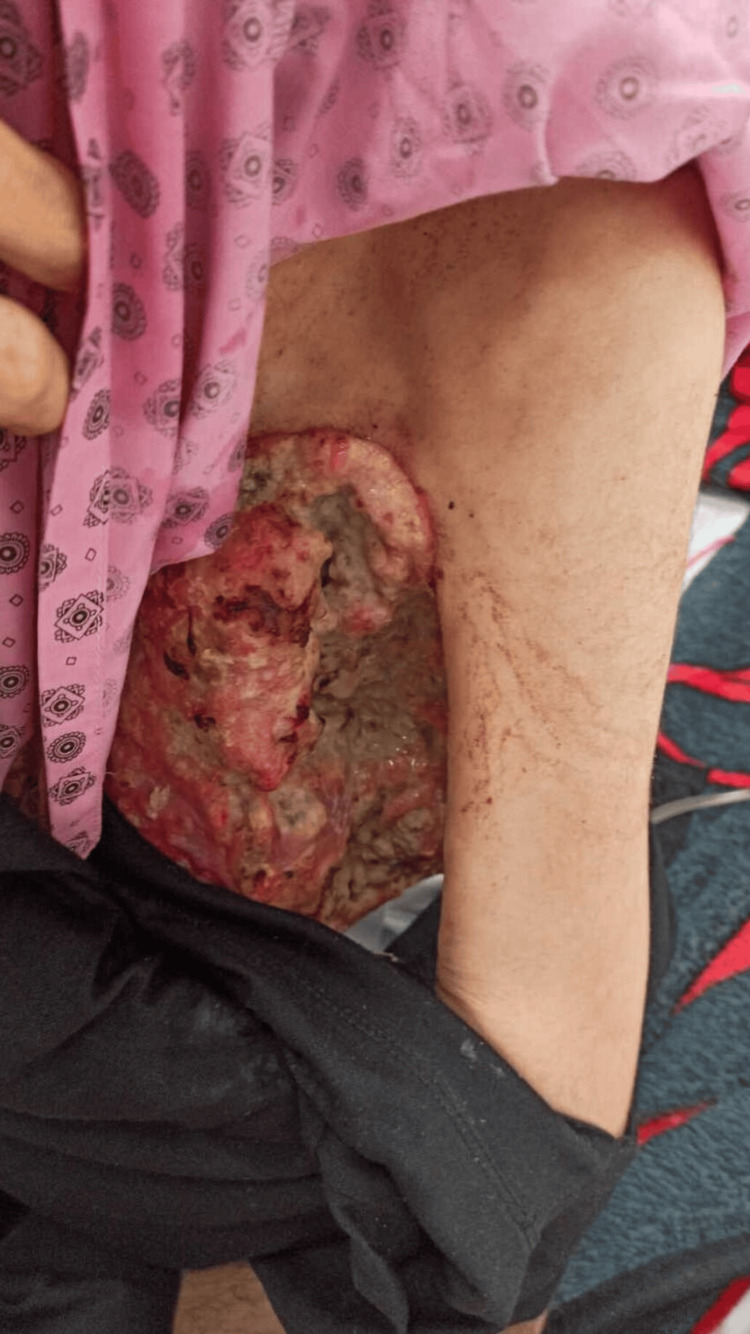
Tumor in left breast (in the left breast, there was a necrotic 17-13 cm mass with clear, ulcerative areas and exudative, malodorous, draining discharge).

**Figure 2:**
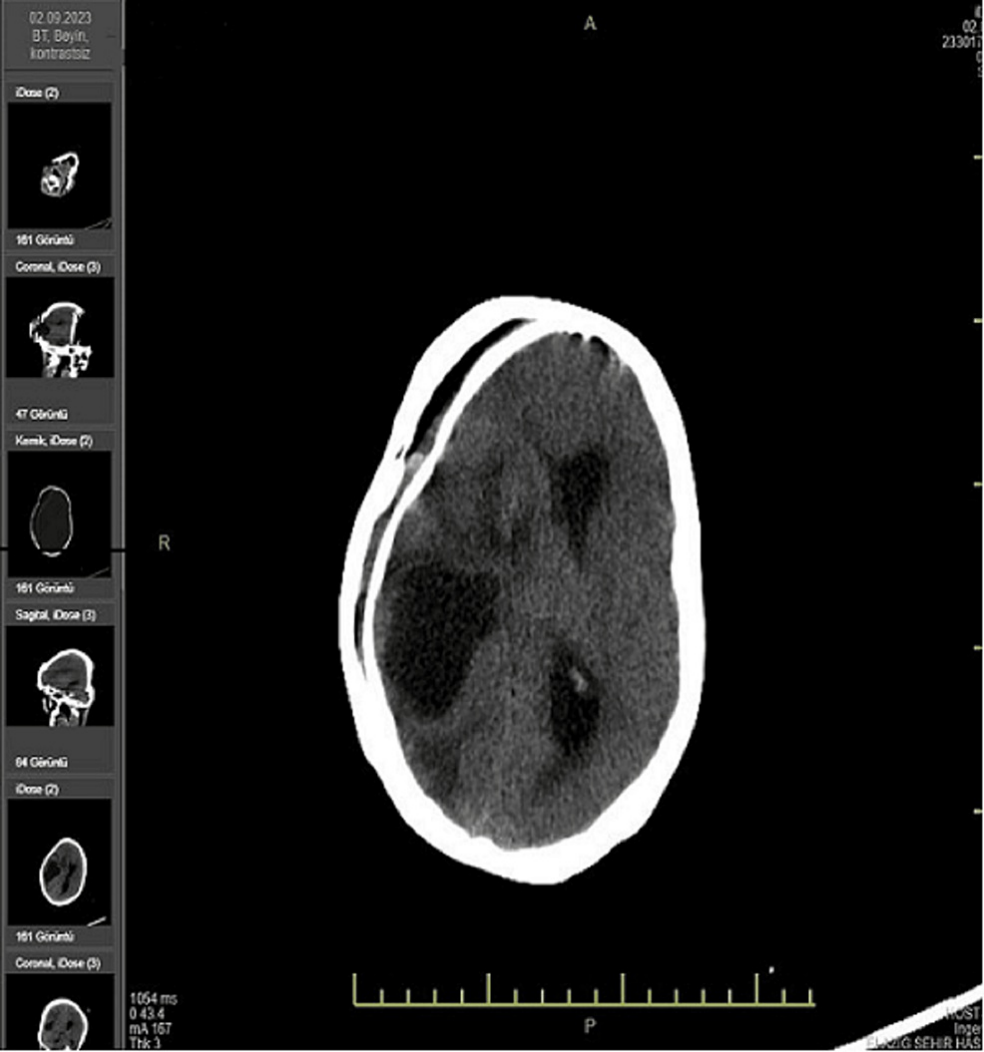
Metastatic tumor in the brain (5.5 cm cystic space-occupying lesion in the right occipitotemporal region and surrounding edema)

**Figure 3:**
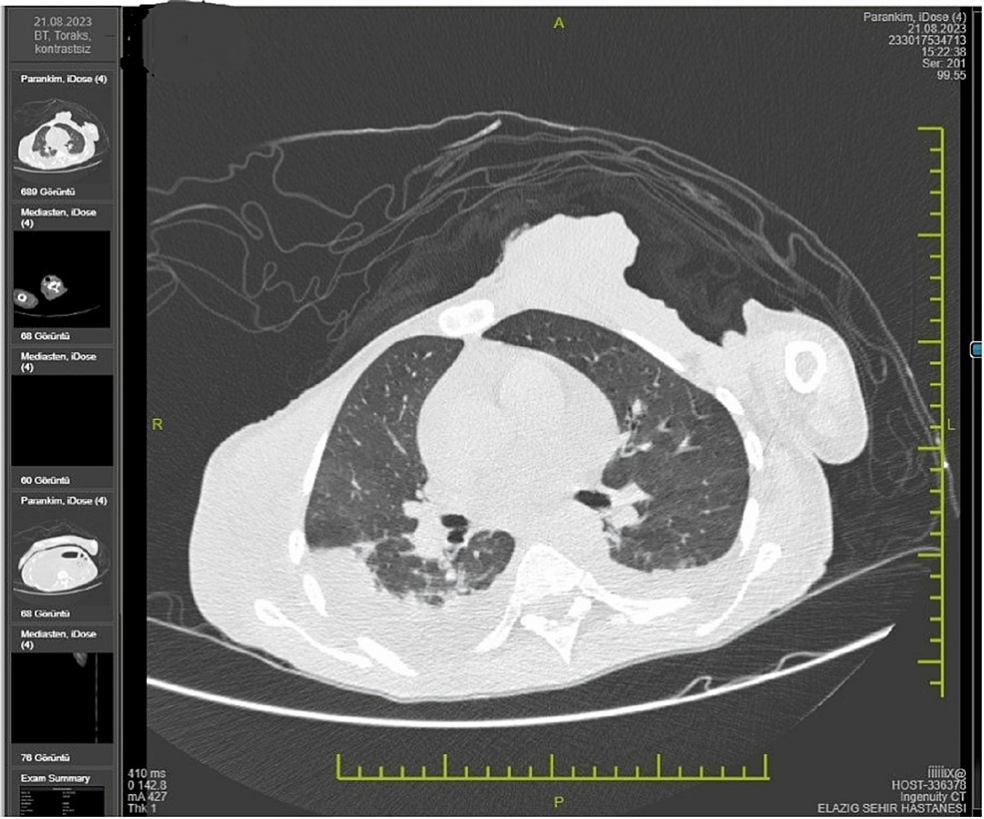
Tomography of the patient (pleural and pericardial effusion and pneumonia)

A 44-year-old female patient, who had never received psychiatric treatment before, called her relatives saying 'an insect bit my breast' and was found naked and wrapped in a blanket by her relatives. At the time, the patient could hardly answer the questions and looked very weak. When the patient's relative asked 'Why do you smell so bad?', the patient replied 'Do you like my perfume'. The patient was then brought to the emergency room. On physical examination, consciousness was clear, scleral icteric, fever: 38 Celcius degrees, pulse: 90, blood pressure 100/80 mmHg, oxygen saturation: 96%, respiration was spontaneous, lung sounds were decreased bilaterally, rales were present in places and heart sounds were deep. The left breast had a necrotic 17-13 cm mass with clear, ulcerative areas and exudative, malodorous, draining discharge (Figure 1). As a result of neuropsychological evaluation and physical examination, the diagnoses of epilepsy, dementia, and encephalitis were excluded. The patient partially responded to the questions and a brain tomography revealed a 5.5 cm cystic space-occupying lesion in the right occipitotemporal region with edema around it (Figure 2). Hemoglobin: 4 g/dL (12-16), white blood cell: 12000 (3.6-11.000), glucose: 71 mg/dL (74-100), albumin: 15 g/DI (15-52), total protein: 50 G/dL(66-83), lactate dehydrogenase: 336 U/L(125-248), C reactive protein: 75 mg/dL (<8) other parameters were within normal range. The patient was hospitalized in the general internal medicine intensive care unit to determine the etiology of general condition disorder, anemia, high infectious value, and masses in the breast and brain. Nutritional support was provided and pleural and pericardial effusion and pneumonia were detected (Figure 3). Infectious diseases were consulted due to elevated infection parameters, pneumonia, and necrotic lesions in the breast. Ampicillin 8 g/per day intravenous treatment was started. Neurosurgery was consulted because of the mass in the brain and anti-edema treatment was started by neurosurgery. 

On the third day of hospitalization, insomnia, introversion, refusal to eat and drink and strange conversations continued and psychiatry was consulted. In the anamnesis taken by the psychiatrist, it was learned that the patient lived alone at home and had complaints of self-talk, seeing visions, talking to herself, insomnia, reluctance, and introversion which started after intense intrafamilial stress factor. When called by her relatives, the patient stated that he had not left the house for more than three months, but he was constantly going on vacations with her husband and child and did not accept guests at home. In the psychiatric examination performed by the psychiatrist, consciousness was clear, oriented, coherent and semimutism, negativism, auditory hallucinations, social withdrawal, disorganized speech, and insomnia were detected. Her family history was unremarkable. Brain metastasis, paraneoplastic encephalitis, dementia, epilepsy, depression, and schizophrenia were considered as preliminary diagnoses. Considering the anamnesis, mental examination, and medical condition, a diagnosis of psychotic disorder was considered according to the Diagnostic and Statistical Manual of Mental Disorders 5th edition [9]. Considering her general medical condition, olanzapine 2.5 mg/day was started and gradually increased. On the third day of psychiatric treatment, the patient started to talk, her hallucinations decreased, her sleep improved and her appetite improved. On the recommendation of neurosurgery and infectious diseases, the oncology clinic was consulted by the internal medicine physician for the etiology of the mass. Biopsy and positron emission tomography were performed upon the recommendation of the oncology physician. On the seventeenth day of hospitalization, metastatic invasive breast cancer was diagnosed as a result of pathologic evaluation. While the patient was on olanzapine 10 mg/day, her psychotic state improved considerably, but the patient was transferred to the oncology clinic in order to organize the malignancy treatment.

## Discussion

We know that patients with psychotic disorders are sometimes alienated from themselves, but it was quite surprising that she did not notice the large and malodorous mass in her breast like the patient mentioned above. We thought that anosognosia, lack of insight, denial could be options. We believe that although rare, this condition can be seen, underlying psychiatric problems may be the cause of late-recognized cancers, and clinicians should be careful in this regard.We believe that although rare, this condition can be seen, underlying psychiatric problems may be the cause of late-recognized cancers, and clinicians should be careful in this regard.

In the literature, other cases in which cancer and psychotic disorders were diagnosed together have also been reported. A 46-year-old male patient with complaints of hearing voices, incoherent thinking and then committing murder was diagnosed with a schizophrenia-like psychosis due to a mass in the temporal lobe [10]. The primary cancer in this case was brain cancer and the behavior differs from our case in this respect. A case of chemotherapy-induced acute psychosis was reported in a female patient with malignant germ cell tumor due to her very irritable, paranoid thoughts, auditory hallucinations and thoughts that she was rich and close to the prime minister during chemotherapy treatment [11]. Unlike our case, this case did not benefit despite using olanzapine 20 mg/day, and it was said that he dramatically benefited when he switched to risperidone. Again, psychotic state secondary to chemotherapy was reported in a patient with invasive ductal breast carcinoma. In this female patient, in addition to the explanation that antipsychotic treatment was started late, it was reported that paliperidone was started when no benefit was seen from olanzapine and risperidone and that the patient benefited and this drug was used during chemotherapy [12]. Since antipsychotic treatment in schizophrenia patients leads to significant differences in inflammatory gene expression and chronic inflammatory responses help cell mutation and cancer development [13], it is thought that breast cancer is more likely to occur in these patients [14]. However, since the diagnosis of psychotic disorder was not known until the diagnosis of breast cancer in our case, our patient was not using antipsychotic drugs.

Various differential diagnoses were considered for this patient. Due to the metastasis in the brain, we thought that it might be an organically induced psychotic disorder. Space-occupying lesions in the brain may lead to personality changes, affective disorders and psychotic disorders. In 18% of cranial masses, the first symptom of the disease is psychiatric behavioral changes [15]. However, when both the patient's clinic and other findings are evaluated together, we understand that the psychotic process started first and then the patient ignored the mass in her breast and presented to us with metastasis. There is a possibility that the metastasis to the brain may have worsened the psychiatric clinic.

Due to the organic instability in the patient, it was thought that the current psychiatric diagnosis might be delirium. Delirium frequently develops in patients with advanced cancer [16]. However, there was no clouding of consciousness in our case, and despite the information that the patient's clinical condition would improve when the underlying cause known for delirium improved, the patient's mental state did not improve until olanzapine was started, although blood values started to improve. Since our patient did not meet some criteria [9] such as duration of illness and delirium, the diagnosis of schizophrenia was excluded.

It is known that there is a relationship between schizophrenia and breast cancer and that schizophrenia has a phenotypic and genetic positive association with breast cancer based on data from a Swedish population-based study [17].

## Conclusions

As a result, cancer can occur in psychiatric patients as in all people. However, in our study, we could not find a case in which the large mass in the breast was not recognized due to the diagnosis of psychosis, and the diagnosis of cancer was delayed and reported with metastasis clinic. We think that it would be beneficial to perform a detailed psychiatric examination in routine cancer screening and to closely monitor patients with mental pathology, especially psychotic patients whose ability to evaluate reality may be impaired.
